# Supplementary material for: Incorporating social knowledge structures into computational models
Source: Nat Commun. 2022 Oct 20;13:6205. doi: 10.1038/s41467-022-33418-2 (PMC9584930; doi:10.1038/s41467-022-33418-2)
Supplement: Supplementary file 1 — Supplementary Information [file 41467_2022_33418_MOESM1_ESM.pdf]

Incorporating social knowledge structures into computational models

Supplementary Information

Koen M.M. Frolichs[1,2], Gabriela Rosenblau[3], and Christoph W. Korn[1,2]

[1] Institute for Systems Neuroscience, University Medical Center Hamburg-Eppendorf, Hamburg, Germany.
[2] Section Social Neuroscience, Department of General Psychiatry, University of Heidelberg, Heidelberg, Germany.
[3] Department of Psychological and Brain Sciences, George Washington University, Washington DC, USA.

Supplementary Figures

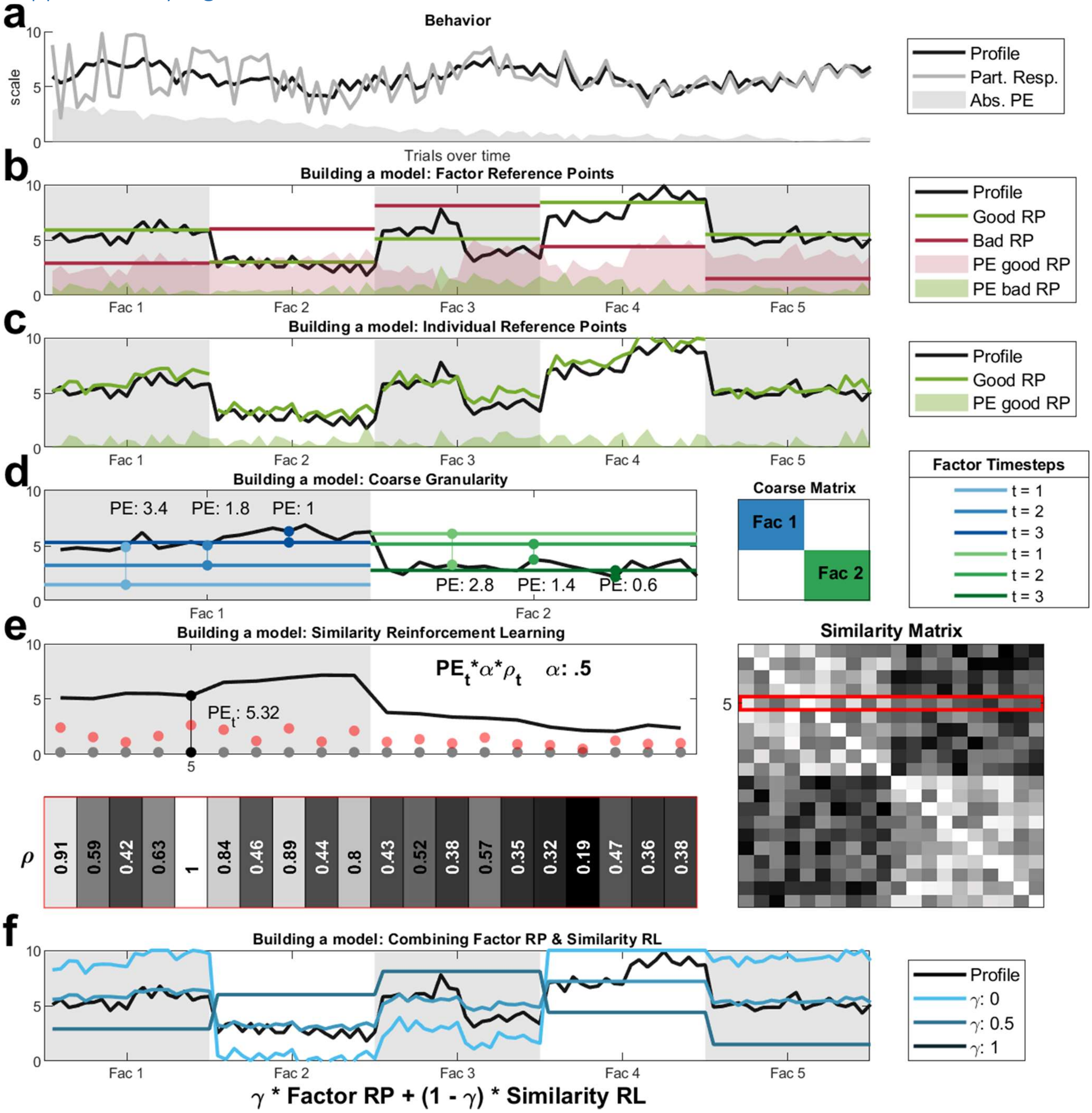

Supplementary Figure 1. Highlighting the main parts of our modelling approach. All data is simulated and solely intended for visualization purposes.

a) Behavioural Data. An idealized learning response pattern over time. The black line represents to-be-learned values on a given task (e.g., self-ratings on personality traits by a certain person). The grey line represents a participant's answers trying to estimate these self-ratings. This participant is learning because over time her answers get closer to the outcome (self-ratings). Similarly, this is indicated by the decrease in the absolute prediction error (PE, transparent grey) over time. Using computational models we can attempt to capture such behaviour. In the following sections, we explain several models of differing complexity that all focus on different pieces of information regarding the self-ratings.

Building a model

In general, the black lines in **b-f** represent self-ratings on personality traits. A real-life experiment would have randomly ordered items but we have ordered them based on five arbitrary factors for easier visualization, where each factor consists of multiple trials. Shaded (grey and white) backgrounds indicate the factors and coloured shaded regions in the lower parts of the plots indicate the PEs of the models with the same colour.

b) Factor Reference Points. A simple model could make use of only a Reference Point (RP) per factor (i.e., a person is represented as having a single mean value per factor). Added are two such RPs, one that fits the data reasonably well (green) and one that does not fit the data well (red). The shaded area at the bottom of the plot represents the absolute PE for both RPs. The worse fit of the red RP is indicated by higher values of the absolute PE. Because this model is overly simplistic we did not include it in our model set.

c) Individual Reference Points. A more complicated model could have RPs for each trait separately since this can capture idiosyncrasies within factors e.g., in Factors 3 and 4 these RPs capture that there is a group of higher and lower rated items. Something that is not present in the previous Factor RP model. In our model set Model 1 [No Learning] captures these individual RPs.

d) Reinforcement Learning. Standard Rescorla-Wagner models use the discrepancy, i.e., the prediction errors (PEs), between the expected and actual outcome values to incrementally update the estimate for the next time step. The magnitude of the updating is determined by the learning rate (usually denoted as: $\boldsymbol{\alpha}$). In this figure we depict simulated data of an agent who learns an average value for two factors, i.e., individual items get presented that belong to one of two factors. The participant automatically categorizes these into their respective factor and learns an average value per factor. The horizontal coloured lines are the expected averages for the first (blue) and second (green) factor where shades indicate the time steps in learning. The first estimate for the first factor (blue) results in a PE of 3.4, after updating this estimate again the resulting PE is: 1.8, a third estimation results in a PE of: 1. Estimates in this figure are spaced out for visualization purposes. For a similar visualization reason the factors have been presented in an ordered (instead of an interspersed) fashion. This represents Model 2 [Coarse Granularity] in our model set.

e) Similarity Reinforcement Learning. Rescorla-Wagner models learn only one value at a time, this can be a trait or an average value (e.g., per factor). However, real-life learning happens broadly from few examples. A mechanism that can explain such generalized learning is to update all traits based on how correlated they are to the current trait.

In the top section, the black line represents a subsection of self-ratings on two factors. For visualization sake it is assumed that the participant expects each trait rating to be zero (grey dots on the x-axis). In this example the participant gets feedback on item number five (black dot), which results in a PE of 5.32 (i.e., the difference between the expectation [0] and the outcome [5.32], the vertical black line between the two black dots). Instead of multiplying this by the learning rate and updating solely this item, the participant can update all traits based on their correlation to this current item. The similarity matrix on the right shows the correlations between items (as for all correlation matrices the main diagonal consists of ones and the matrix is symmetric with respect to the diagonal). Since the model is learning about the fifth item, the fifth row (in the similarity matrix) is selected to update all other items. On the bottom, this row has been enlarged and correlation coefficients have been added. Each item is then updated by multiplying the PE, the learning rate, and the correlation coefficient resulting in the new estimates (red dots, top section). Item 5 is updated according to the standard RL. While only receiving feedback about item 5, updates occur for all other correlated items in the set. Items for which the correlation is (close to) zero are barely updated because learning about item 5 gives no or very little information about uncorrelated items. In our model set this is Model 4 [Fine Granularity]. Using this approach one could recreate the functionality of the coarse models by using a similarity matrix with 1's within a factor and 0's outside.

f) Combining Factor RP & Similarity RL. Using a weighting parameter (γ) we can combine the previously discussed models into combination models. The shaded blue lines are several weighted combinations of the Factor RP and the Similarity RL. Notably, if the weighting parameter is either 0 or 1 it is simply one of the subcomponents (Factor RP or Similarity RL) of this combination model. In this simulated example, equally weighting both subcomponents (γ: 0.5) most accurately follows the self-ratings and thus would be the best fitting model. Akin to the learning rate, the $\boldsymbol{\gamma}$ is usually a free parameter that is estimated during model fitting. This approach is used for Model 3 [Coarse Granularity & Population RP] and Model 5 [Fine Granularity & Population RP].

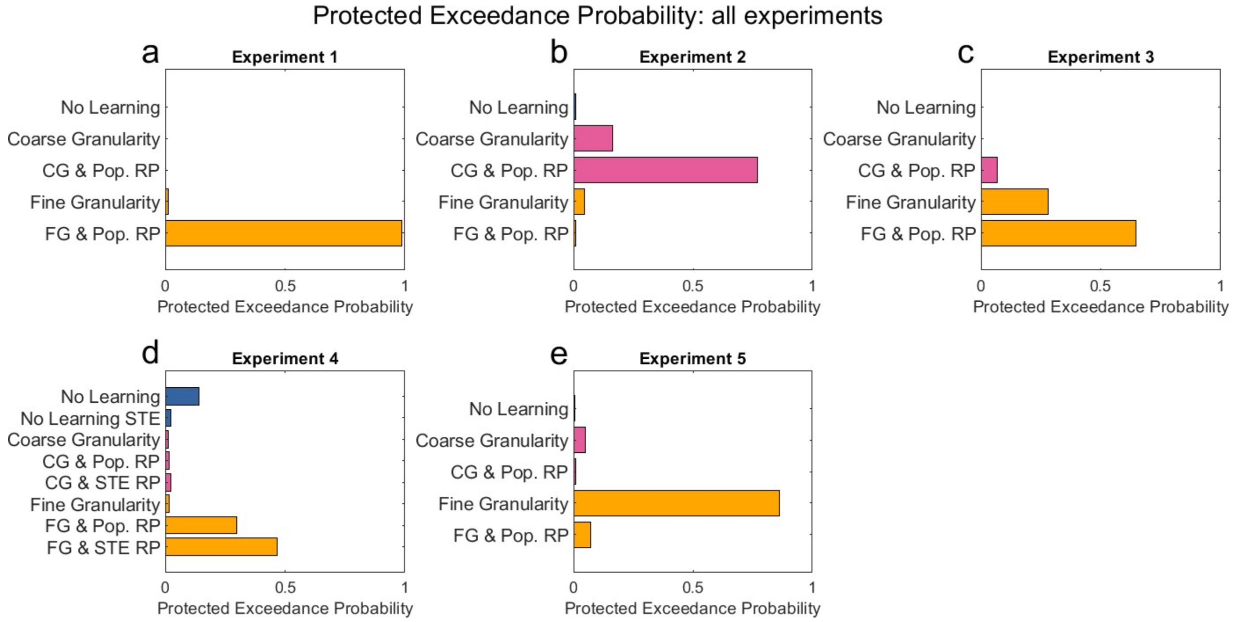

Supplementary Figure 2. Protected exceedance probabilities for all experiments.
Model selection is performed using random-effects (RFx) analysis
a) Consistent with fixed-effects (FFx) analysis (depicted in Figure 3a), Model 5 [Fine Granularity & Population RP] was the best fitting model (n=35).
b) Consistent with fixed-effects (FFx) analysis (depicted in Figure 4a), RFx-analysis indicated Model 3 [Coarse Granularity & Population RP] to be the best model (n=41).
c) Model comparison indicated that model 5 performed best according to FFx (Figure 5a) and RFx analyses (n=59).
d) Models using a stereotypical RP have a darker colour than their "twins" with the standard RP. Model comparison evidence is low for Experiment 4, with RFx analysis being slightly ahead for model 8 [Fine Granularity and Stereotypical RP] (n=29).
e) Model comparison indicated Model 4 as the best model according to both FFx (Figure 7a) and RFx analyses (n=28).

CG = coarse granularity, FG = fine granularity, RP = reference point, # = number of, PEs = prediction errors, SEM = standard error of the mean.

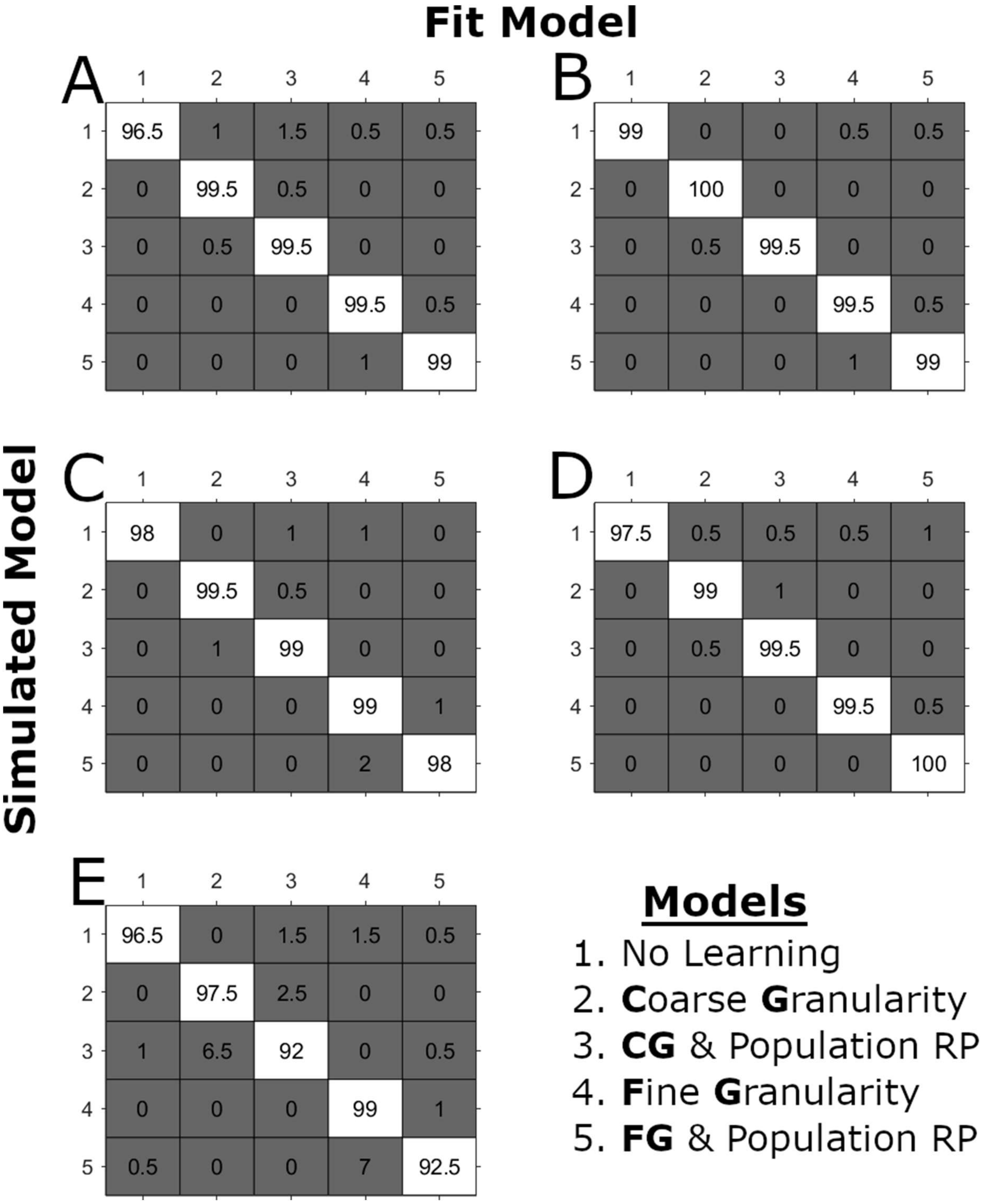

Supplementary Figure 3. Confusion matrices for every experiment.

To test whether data created with a model would also be best fitted by that model, a confusion matrix was created for every experiment separately by randomly sampling parameter values 200 times. With these random parameter values, data was simulated and noise, sampled from a standard normal distribution, was added. After this, the models were fitted to the simulated data. Ideal recovery is reflected by the identity matrix (i.e., all values on the diagonal being 100%). Model recovery for all experiments indicated that our models were recovered correctly in most cases (>92%), i.e., models were distinguishable. **A-E** represent experiments 1-5.

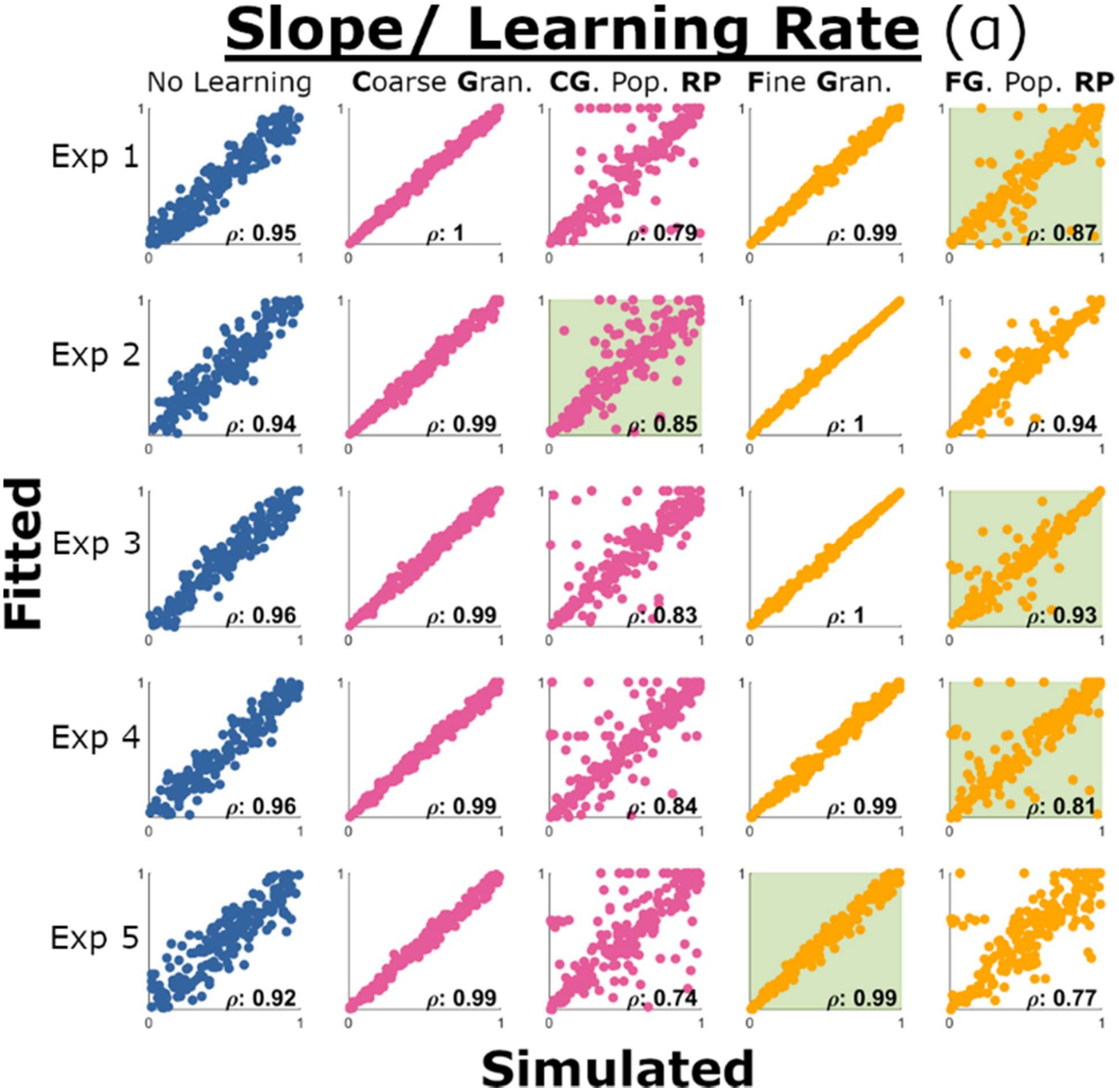

Supplementary Figure 4. Parameter recovery for alpha (learning rate).
To check whether model fitting returns meaningful parameter values, we performed parameter recovery on all models and all experiments separately. We simulated data using random parameter values, to which noise was added. After this, all models were fitted to this simulated data and parameters recovered. We measured recovery by calculating the Pearson's correlation coefficient (ρ) where a value closer to 1 indicated better recovery. Models are ordered along the columns, every row is a separate experiment. For Model 1 (first column, blue dots) we looked at the regression slope and for all the other experiments at the learning rate (α). Parameter recovery for all experiments and all models was well within acceptable bounds. Green boxes indicate the winning model for that specific experiment. For Experiment 4, we only added the standard 5 models, choosing to omit the stereotype models. This was done because the stereotype models are functionally the same when it comes to simulated data and thus would not add any information.

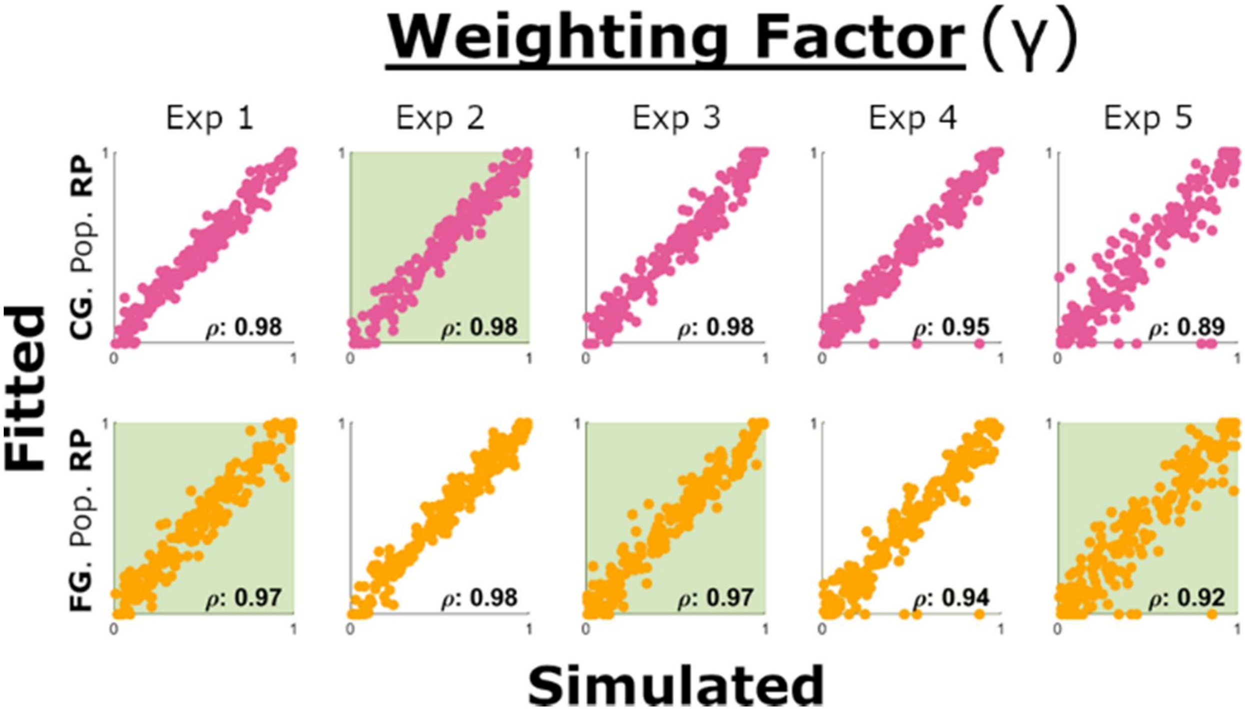

Supplementary Figure 5. Parameter Recovery for gamma (weighting factor).
See **Supplementary Figure 4**, for an explanation of how we conducted parameter recovery. The Gamma parameter was only used in Model 3 [Coarse Granularity and Population Reference Point] and Model 5 [Fine Granularity and Population Reference Point]. Models are ordered along the rows and experiments on the columns. Recovery for all models and all experiments is good across the whole range of values. Green boxes indicate when a model was the best fit for that specific experiment.

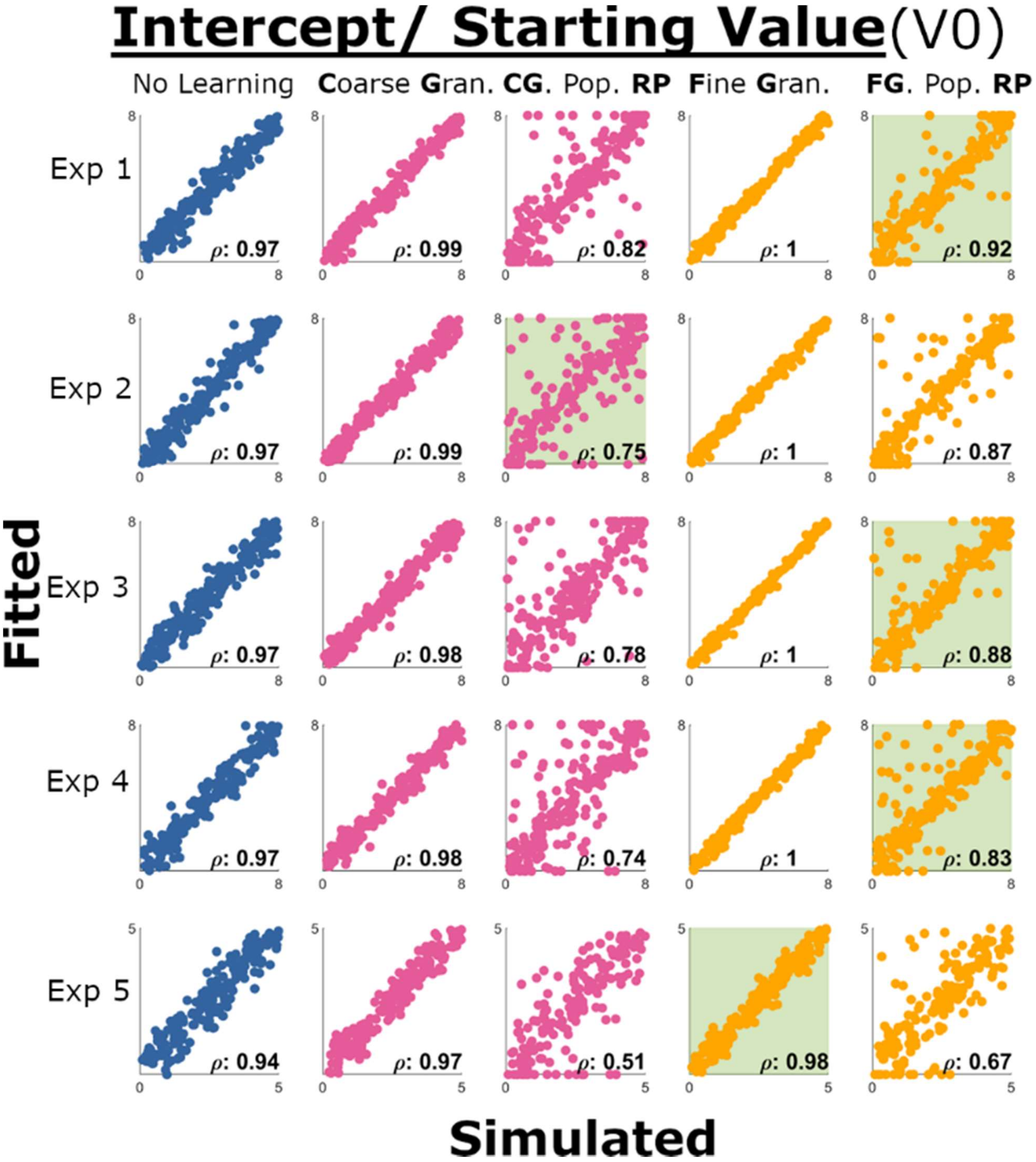

Supplementary Figure 6. Parameter recovery V0 (starting value).
See **Supplementary Figure 4**, for an explanation of how we conducted parameter recovery. Parameter recovery for the intercept (Model 1) and the starting value (Models 2-5) is acceptable for all models. Every row is an experiment and each column is a different model, green boxes indicate this was the winning model for that experiment.

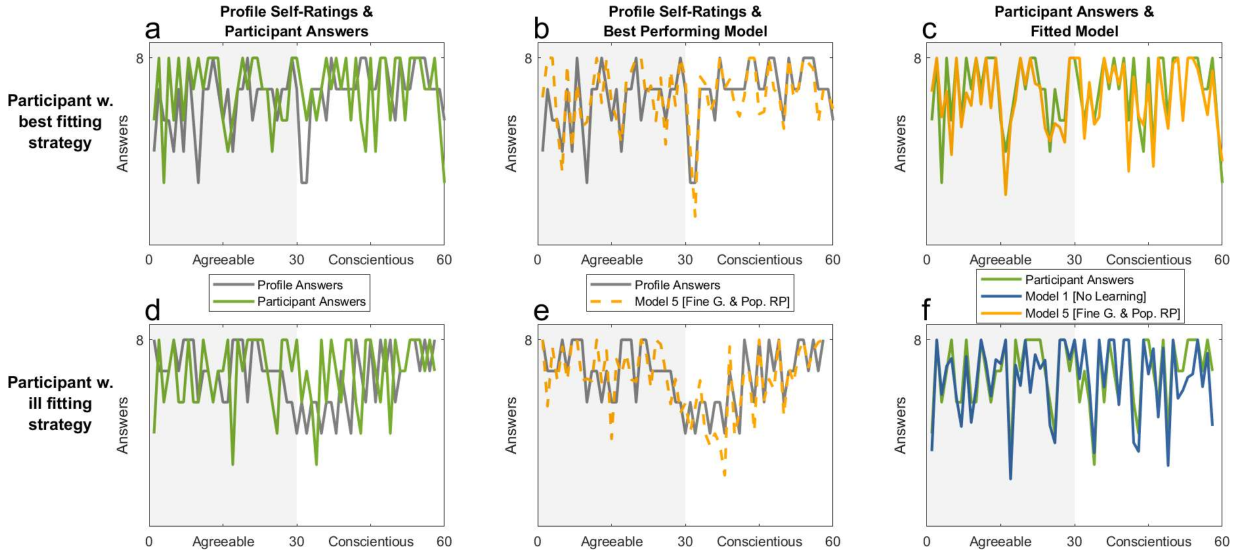

Supplementary Figure 7. The rationale of the best performing models (in the set).
Data in this figure are selected from two participants (top and bottom row) from Experiment 2. Data have been sorted for trials and factor i.e., items have been sorted based on when they were encountered within the factors agreeableness and conscientiousness.

The left panels (a, d) show the profile answers in grey (this is the feedback participants received) and participants' answers are shown in green. During model fitting, models are fitted on these participant answers (rightmost panels: c, f), here we see that the top participant's answers are best explained by Model 5 (orange) and the answers by the bottom participant are best explained by Model 1 (blue).

The middle column (b, e) shows the 'best performing models', these are models fitted on profile answers (grey line) i.e., models are fitted as if they have full information about the profiles, like participants had. For both profiles, Model 5 is the best performing model in our set of models.

Comparing the best fitting models with the models that best explained participant data helps us understanding whether participants used an effective strategy or not. In this case, for the top participant, the strategy used is the same as the 'best performing strategy' indicating that the participant used an effective strategy. However, the bottom participant used a different strategy from the 'best performing strategy' which shows that their strategy could still improve.

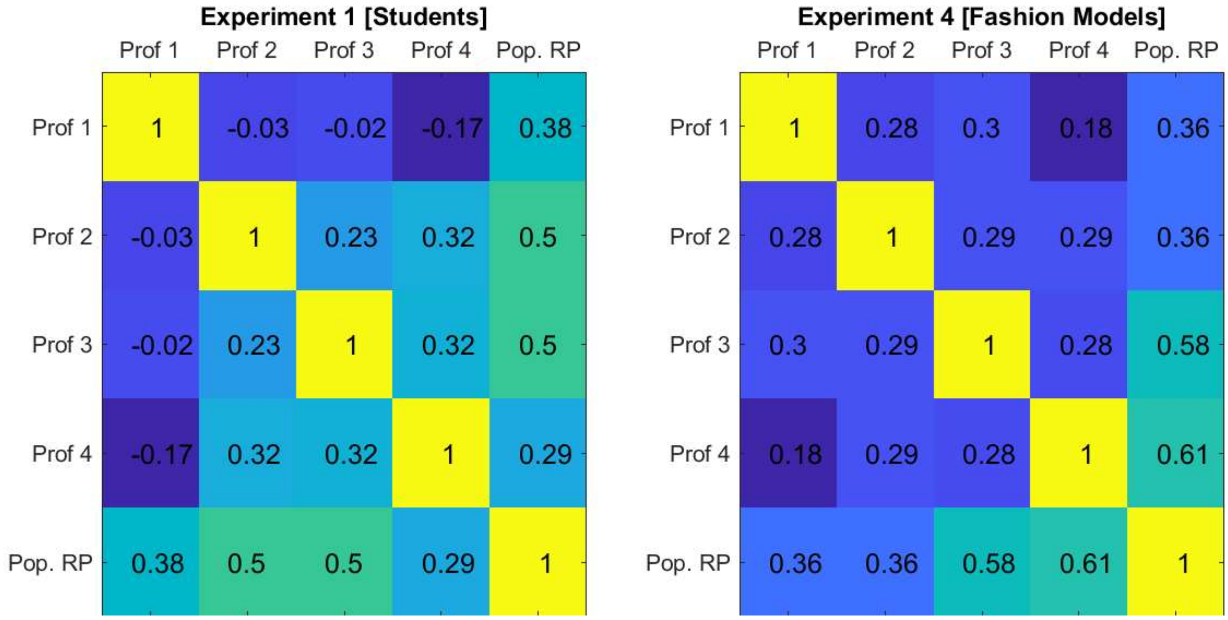

Supplementary Figure 8. Pairwise correlations of profiles and population averages for experiment 1 and 4. The correlation coefficients between profiles and the RP seem to be higher for Experiment 4 than Experiment 1. This additional analysis was performed to understand why the best performing model for Experiment 4 (**Figure 6b**) indicated that the No Learning model was best. One potential explanation is that the fashion model profiles are more correlated with the population RP than the profiles used in our other experiments. Thus an agent performing the task needs less information to do the task efficiently compared with the other experiments. Interestingly enough, participants still seemed to use a complex strategy [Model 5] during this task.

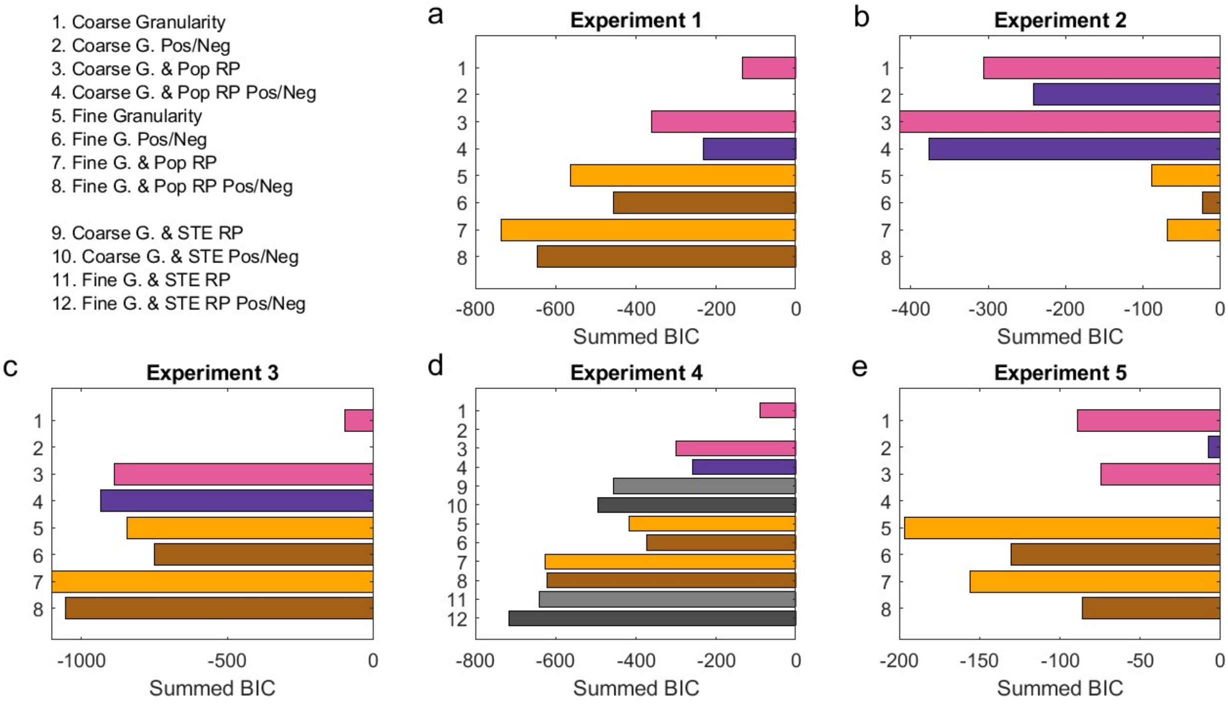

Supplementary Figure 9. Positive/ Negative models (P/N) compared with the standard models.
Positive/ Negative models (Pos/Neg) have two parameters for the learning rate (α) by splitting it up for positive and negative PEs. This allowed us to check whether participants learned differently from positive and negative PEs. Each plot contains the regular models in standard colours. Below each regular model are those that make use of the positive/ negative learning rates (shown in darker colours, i.e., purple for the coarse models and brown for the fine models). For Experiment 4 we also included the stereotype models and their respective stereotype Pos/Neg models (these are depicted in light grey for the standard stereotype models and dark grey for the stereotype models that also use the Pos/Neg learning rates).

CG = coarse granularity, FG = fine granularity, RP = reference point, PEs = prediction errors, STE = Stereotypical

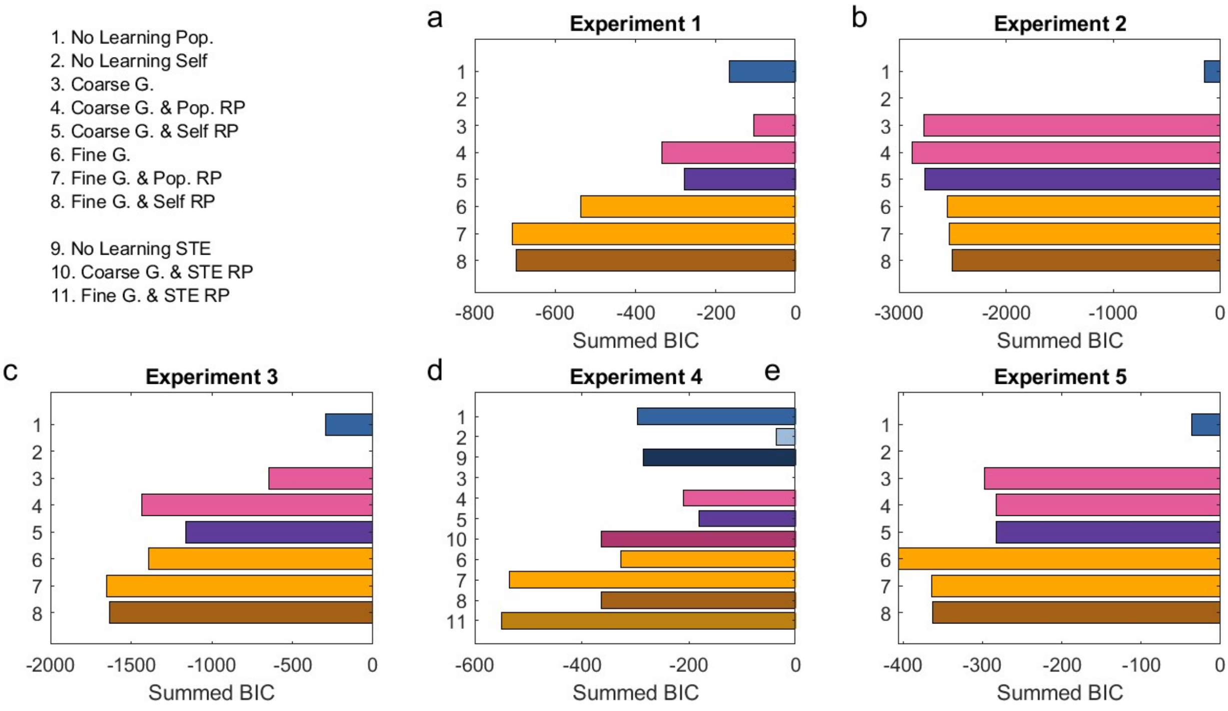

Supplementary Figure 10. Model comparison of all models with additional models that use the participants' self-ratings as reference point (RP) instead of the population RP. The regular models are displayed in their original colours, directly below these are the models that use the Self RPs (in colours: light blue [No Learning], purple [Coarse], and brown [Fine]). For experiment 4 the same order holds but the STE models are added below the Self RP models in their original darker colours (see figure 6). Models that use self-ratings as their RP are always a little worse than those that use the population average RP.

CG = coarse granularity, FG = fine granularity, RP = reference point, PEs = prediction errors, STE = Stereotypical

Supplementary Methods

Optimal Parameter Space

In combination with our simulations for the best performing model (**Figures 3-7 panel b** and **Supplementary Figure 7** for a detailed explanation), we performed an extra analysis in an attempt to visualize the results of the model fitting procedure in more detail.

First, we simulated all models and their respective fits over the whole parameter space on simulated profiles. Since our models had a relatively limited parameter space (i.e., maximum of 3 free parameters), we could explore their parameter space with relatively little computations. Each model's parameter space was mapped through a grid-search on all parameters, this was executed by creating a grid using 100 steps between the parameters bounds (i.e., [0 1] or [1 8]), this meant that the coordinates on the grid corresponded to the parameter settings. These parameter settings were run for every dataset separately using the procedure from the best performing models (where the fit was determined using the sum of squared errors). Models 1, 2, and 4 have 2 free parameters and thus result in 2-dimensional plots, whereas Models 3 and 5 (panels: c, e) have 3 free parameters. To display Models 3 and 5 in a similar manner to the other models, we took the best fitting "slice" in the z-axis dimension (i.e., for the weighting parameter γ).

On this parameter space (where each axis represents distinct values on a parameter), we superimposed each participants' fitted parameters (grey diamonds for models that best fit participants behaviour and white dots for the worse fitting models) to check if fitting procedures performed correctly and how the fitted parameters behaved for every model.

Finally, we expected that visualizing the data in this manner would make it easier to catch a-priori unexpected ways that the models could fit the data (e.g., by ignoring one parameter completely).

These visualizations indicated that fitting procedures went well for all models (e.g., most fitted parameters fell within the optimal range) and all experiments (**Supplementary Figure 11-15**).

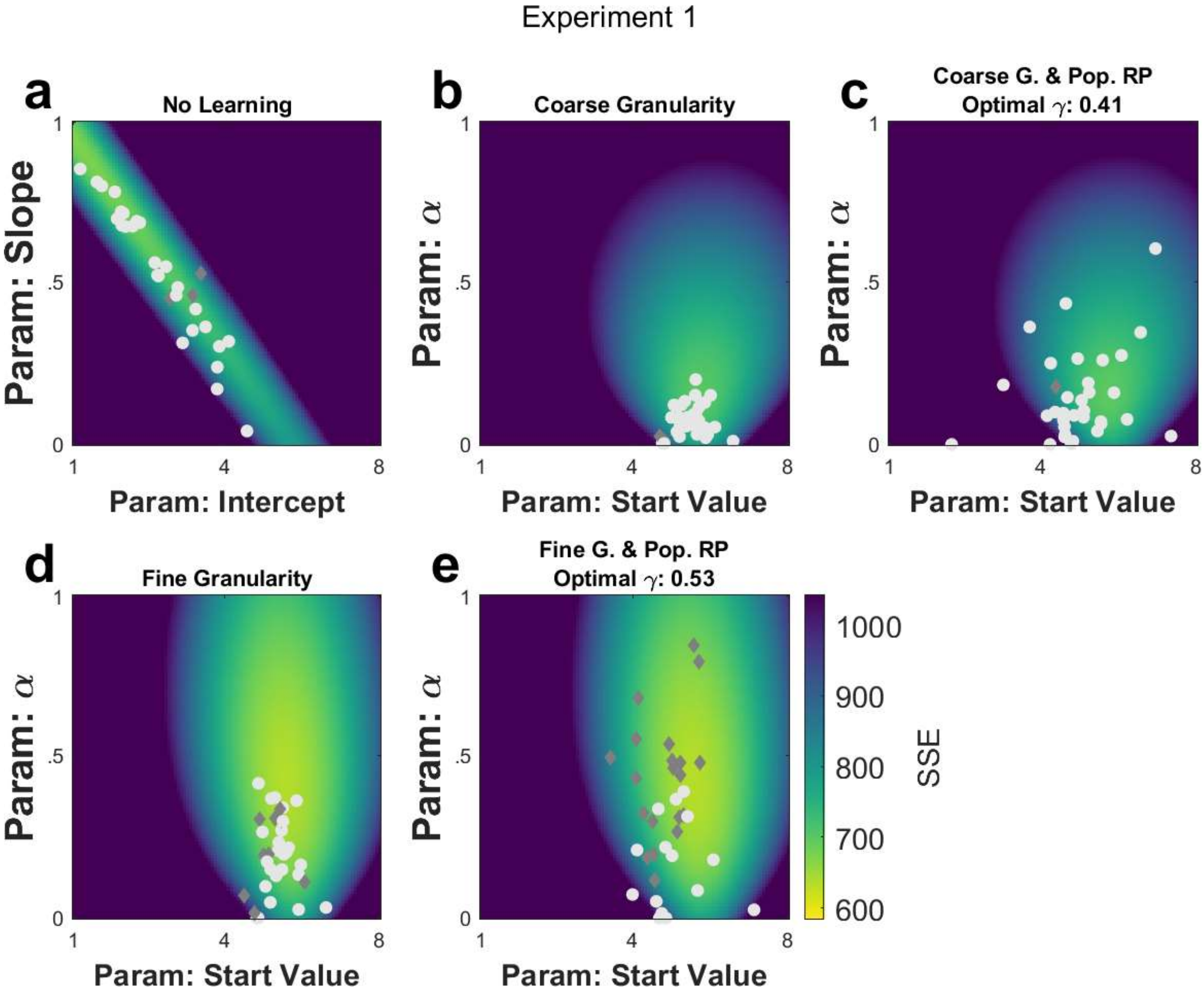

Supplementary Figure 11. Optimal parameter space with fitted parameters from participants in Experiment 1. There is a large overlap between the optimal simulated sum of squared errors (SSE) (light green) and participants' best fitting parameters (grey dots (ill-fitting model for this participant) and diamonds (best fitting model for this participant)). Because Models 3 and 5 used three free parameters their parameter space cannot be displayed on a Cartesian plane, therefore we chose the slice with the "optimal" gamma for these models (c, e). Best fitting model was Model 5 (panel: e). These plots show that the model fitting procedure went as expected.

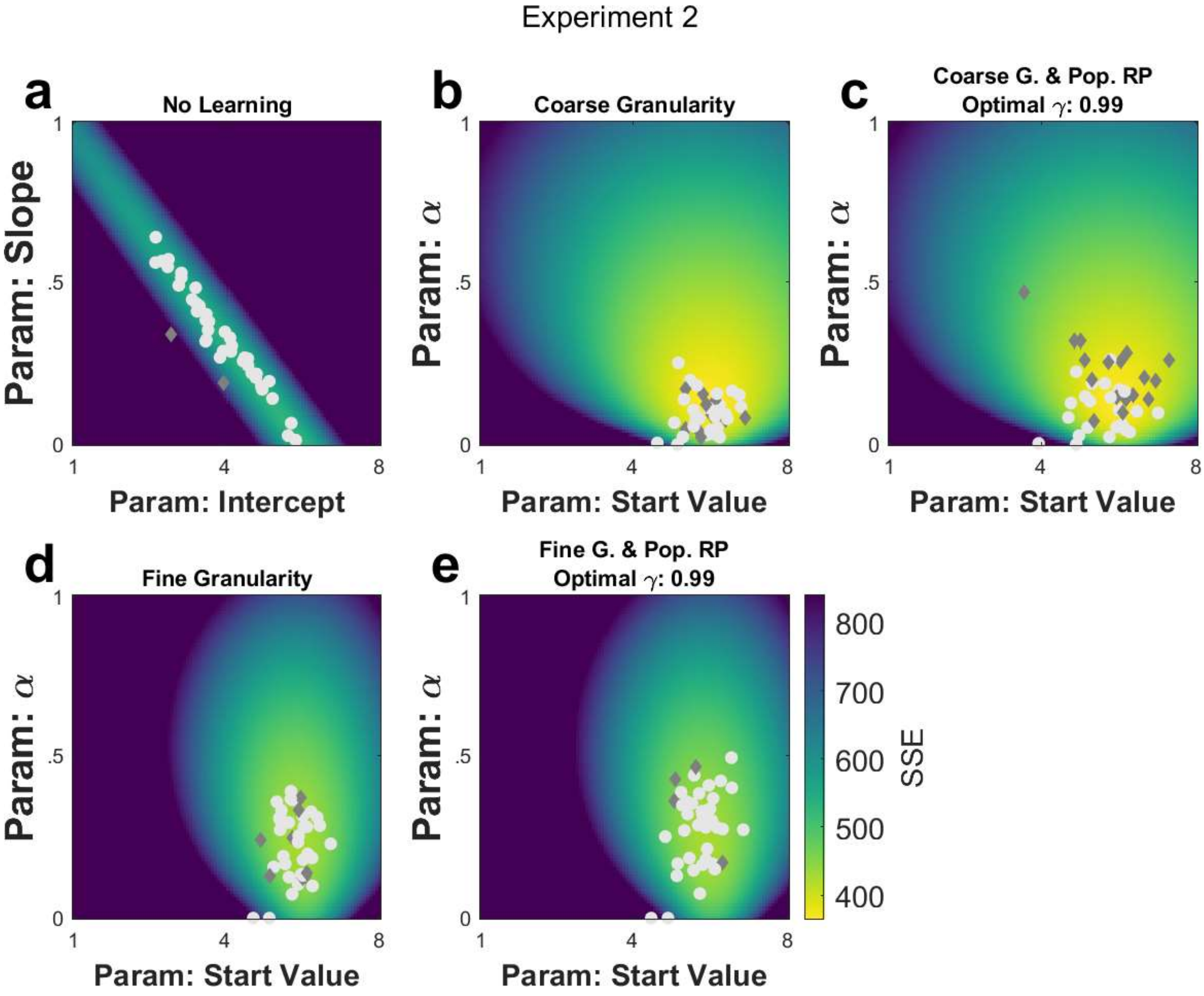

Supplementary figure 12. Optimal Parameter Space for Experiment 2.
Same logic as **Supplementary Figure 11**. All parameter estimates fell within the optimal range for each model with the model fitting procedure performing as expected. Best fitting model was Model 3 (panel: c).

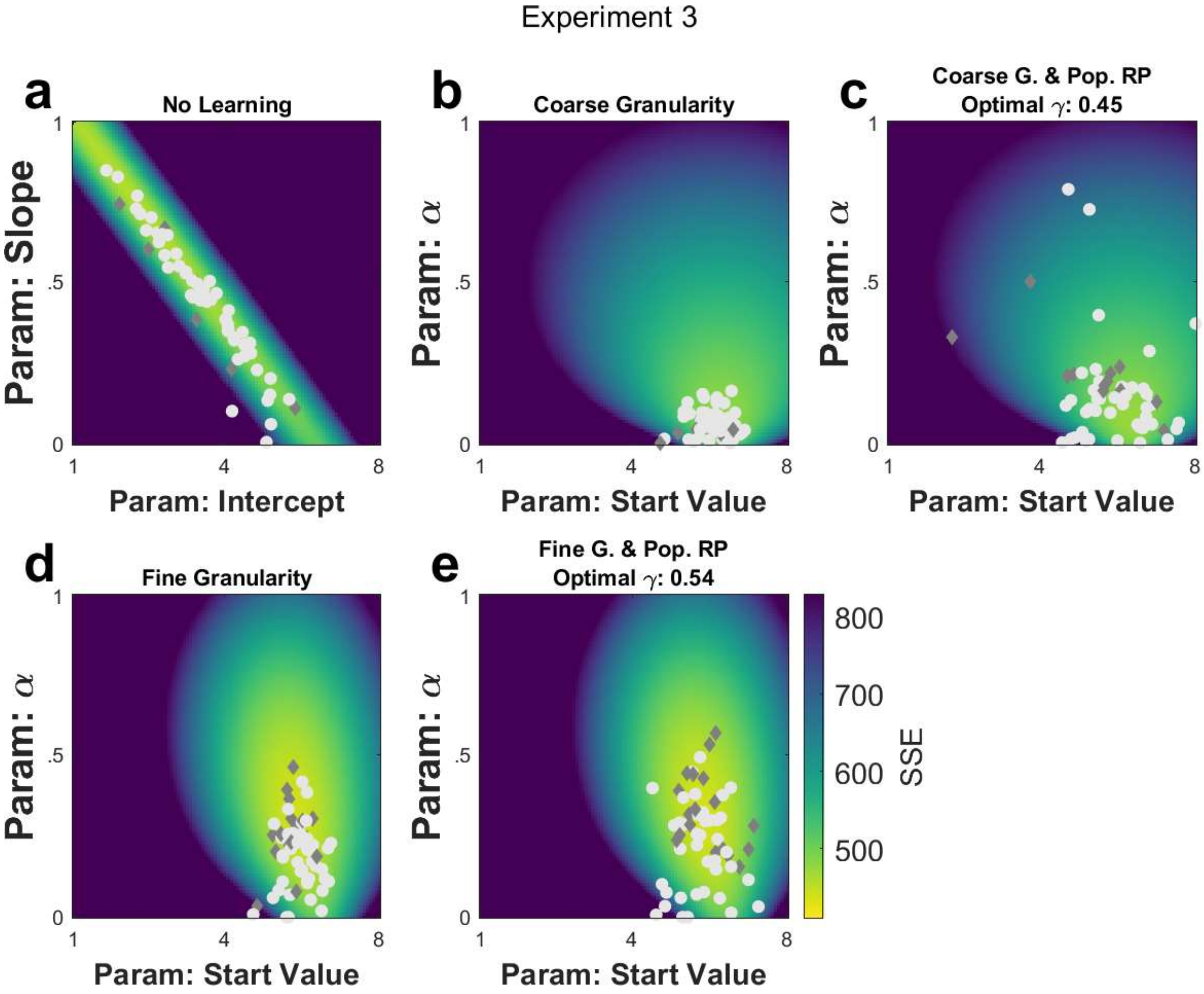

Supplementary figure 13. Optimal Parameter Space for Experiment 3.
Same logic as **Supplementary Figure 11**, best fitting model was Model 5 (panel: e). Parameters fell within the optimal range for all models.

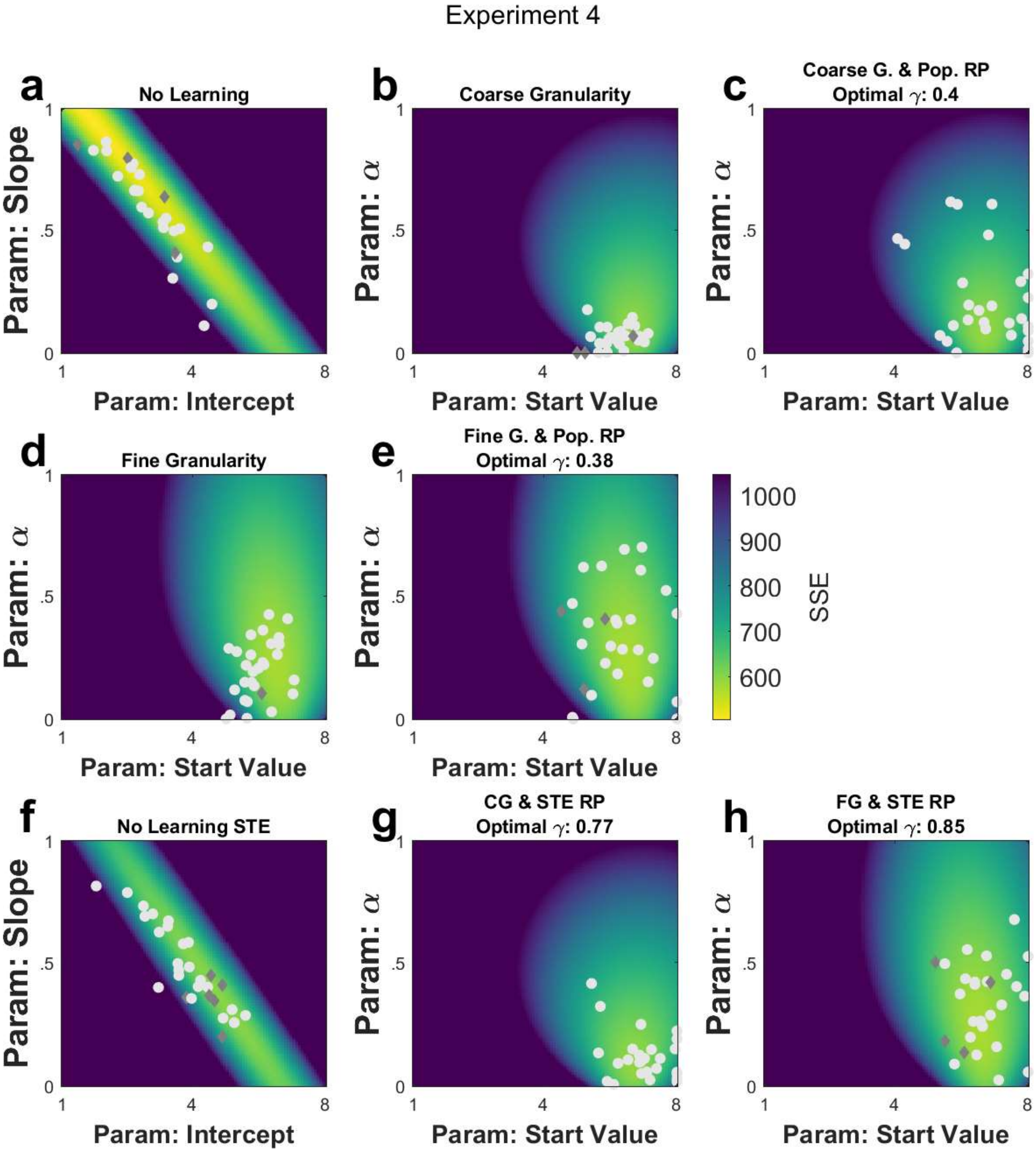

Supplementary figure 14. Optimal Parameter Space for Experiment 4.
Same logic as **Supplementary Figure 11**, best fitting model was Model 8 (panel: h). Experiment 4 had three additional (stereotypical) models. That is, Models 2, 5, and 8 (panels: f-g) in this figure were functionally the same as Models 1, 4, and 7 (panels: a, c, and e) with the exception of their reference points. Evidently from the plots, this change in RP did not have a major influence on the fitting procedure.

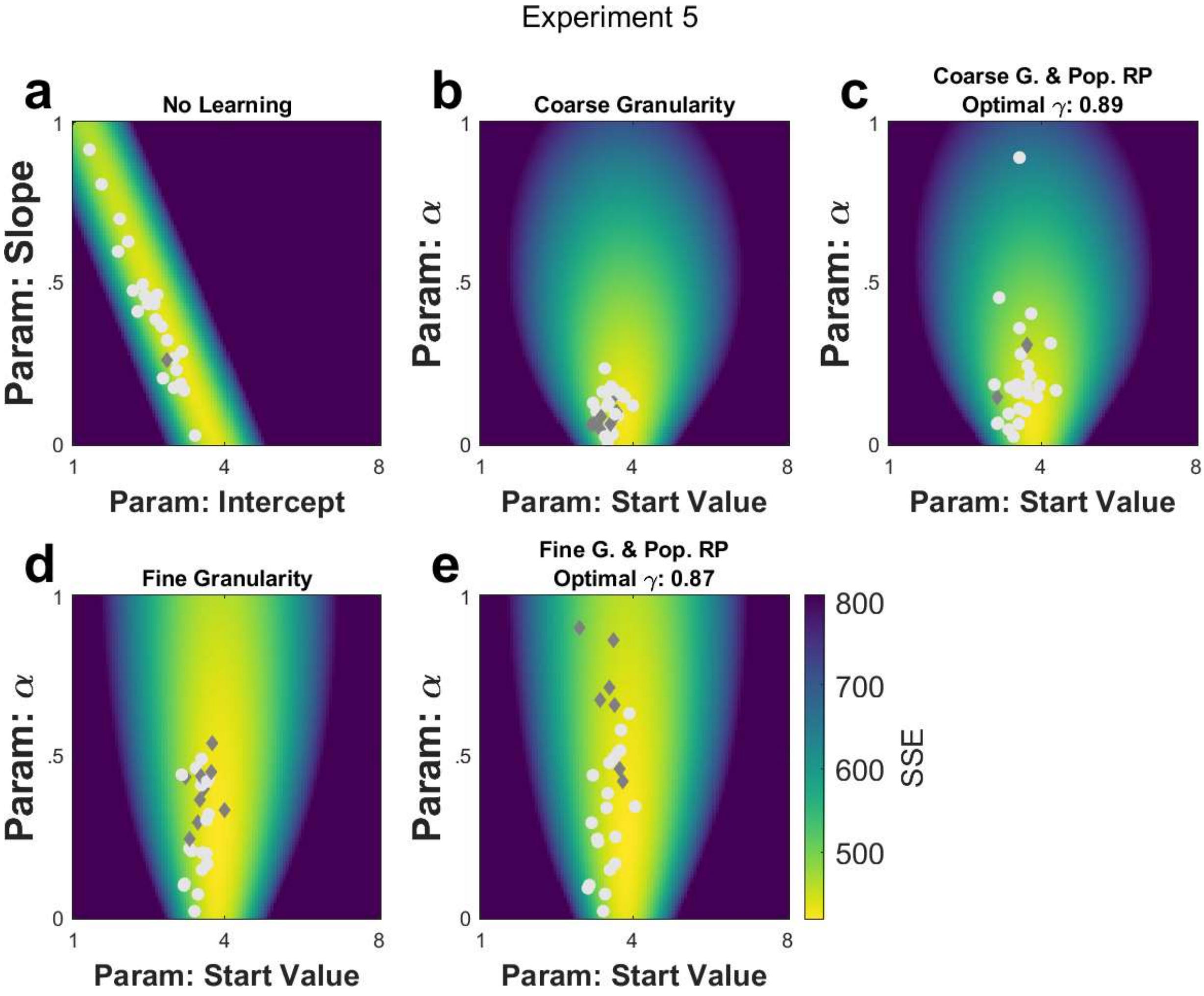

Supplementary figure 15. Optimal Parameter Space for Experiment 5.
Same logic as **Supplementary Figure 11**, best fitting model was Model 4. Model fitting for experiment 5 resulted in a lower absolute SSE than the other experiments. This might have been caused by the smaller answer scale ([0 5] compared to [0 8]). Model fitting procedure went as expected.

Supplementary Tables

Table S1. Stimuli used in Experiments 1 & 4

Item number	German trait word used	Approximate English translation
**Factor 1: neuroticism – positive traits**		
**1**	gelassen	composed
**2**	locker	easy-going
**3**	selbstständig	self-reliant
**4**	souverän	confident
**Factor 1: neuroticism – negative traits**		
**5**	ängstlich	anxious
**6**	feige	cowardly
**7**	launisch	moody
**8**	unentschlossen	indecisive
**9**	unsicher	insecure
**10**	wehleidig	whiny
**Factor 2: extraversion – positive traits**		
**11**	enthusiastisch	enthusiastic
**12**	lebenslustig	fun-loving
**13**	schlagfertig	articulate
**14**	tatkräftig	dynamic
**Factor 2: extraversion – negative traits**		
**15**	humorlos	humorless
**16**	kalt	cold-hearted
**17**	scheu	unassertive
**18**	unnahbar	inapproachable
**Factor 3: openness to experience – positive traits**		
**19**	kreativ	creative
**20**	offen	open-minded
**21**	spontan	spontaneous
**22**	tolerant	tolerant
**23**	vielseitig	versatile
**24**	wissbegierig	inquisitive
**Factor 3: openness to experience – negative traits**		
**25**	bieder	overly conservative
**26**	engstirnig	narrow-minded
**27**	träge	lazy
**28**	voreingenommen	biased
**Factor 4: agreeableness – positive traits**		
**29**	einfühlsam	empathetic
**30**	freundlich	friendly
**31**	gesellig	sociable
**32**	großzügig	generous
**33**	hilfsbereit	helpful
**34**	höflich	polite
**35**	respektvoll	Respectful
**36**	vertrauenswürdig	trustworthy
**37**	zuverlässig	reliable
**Factor 4: agreeableness – negative traits**		

**38**	aggressiv	aggressive
**39**	arrogant	arrogant
**40**	egoistisch	selfish
**41**	eitel	conceited
**42**	gehässig	spiteful
**43**	großmäulig	loud-mouthed
**44**	hinterhältig	conniving
**45**	rücksichtslos	inconsiderate
**46**	stur	stubborn
**47**	unsympathisch	unpleasant
**Factor 5: conscientiousness – positive traits**		
**48**	aufrichtig	honest
**49**	bescheiden	modest
**50**	diszipliniert	organized
**51**	effizient	efficient
**52**	fleißig	hard-working
**53**	kompetent	competent
**54**	ordentlich	tidy
**Factor 5: conscientiousness – negative traits**		
**55**	chaotisch	chaotic
**56**	inkonsequent	inconsistent
**57**	leichtsinnig	foolhardy
**58**	pedantisch	pedantic
**59**	unpünktlich	tardy
**60**	zwanghaft	obsessive

The 60 trait adjectives used here were a sub-selection of the 80 trait words used in previous studies (Korn et al., 2012, 2014). Half of the selected words were positive and half were negative (according to ratings in the earlier study; Korn et al., 2012). The trait adjectives were hand-annotated according to the Big-Five categories. These hand-annotations were compared to a larger list of German trait adjectives, which relied on a slightly different factorization than the Big-Five (Ostendorf, 1990). We tried to use English translations consisting of one word. As is often the case for translations, a mixture of words could give a better idea of the word meaning (especially in the case of words with rather similar meaning).

Table S2. Stimuli used in Experiments 2 & 3

Item number	German trait word used	Approximate English translation
**Factor 4: agreeableness – positive traits**		
**1**	altruistisch	altruistic
**2**	anständig	decent
**3**	aufrichtig	honest
**4**	begnügsam	unpretentious
**5**	diplomatisch	diplomatic
**6**	ehrlich	candid
**7**	fair	fair
**8**	freigiebig	bountiful
**9**	generös	open-handed
**10**	gerecht	just
**11**	großmütig	magnanimous
**12**	großzügig	generous
**13**	hilfsbereit	helpful
**14**	kollegial	like a good colleague
**15**	konstruktiv	constructive
**16**	kooperativ	cooperative
**17**	loyal	loyal
**18**	rücksichtsvoll	considerate
**19**	selbstlos	selfless
**20**	solidarisch	solidary
**21**	spendabel	generous
**22**	treu	faithful
**23**	unbestechlich	incorruptible
**24**	uneigennützig	disinterested
**25**	unterstützend	supportive
**26**	verlässlich	dependable
**27**	verständnisvoll	understanding
**28**	vertrauenswürdig	trustworthy
**29**	verzeihend	forgiving
**30**	zuverlässig	reliable
**Factor 5: conscientiousness – positive traits**		
**31**	akkurat	accurate
**32**	anpackend	energetic
**33**	arbeitsam	industrious
**34**	beharrlich	persistent
**35**	diszipliniert	organized
**36**	effizient	efficient
**37**	eifrig	keen
**38**	engagiert	engaged
**39**	entschlusskräftig	decisive
**40**	fleißig	hard-working
**41**	flink	swift
**42**	fokussiert	focused
**43**	geduldig	patient
**44**	genau	meticulous
**45**	gewissenhaft	conscientious

**46**	gründlich	thorough
**47**	konsequent	consistent
**48**	leistungsorientiert	achievement-oriented
**49**	ordentlich	tidy
**50**	ordnungsliebend	orderly
**51**	pflichtbewusst	dutiful
**52**	planvoll	tactical
**53**	pünktlich	punctual
**54**	sorgfältig	diligent
**55**	systematisch	systematic
**56**	tatkräftig	dynamic
**57**	tüchtig	strenuous
**58**	verantwortungsbewusst	responsible
**59**	verantwortungsvoll	responsible
**60**	zielstrebig	goal-oriented

Fifteen of the 60 trait adjectives in this list were selected from the list of 80 trait words used in previous studies (Korn et al., 2012, 2014). All selected words were positive. The trait adjectives were hand-annotated according to the Big-Five categories. We tried to use English translations consisting of one word. As is often the case for translations, a mixture of words could give a better idea of the word meaning (especially in the case of words with rather similar meaning).

Table S3. Stimuli used in Experiment 5

Item number	German sentence used	English translation
**Factor 1: neuroticism / emotional stability**		
**1**	Ich gerate leicht in Stress.	I get stressed out easily.
**2**	Ich bin die meiste Zeit entspannt.	I am relaxed most of the time.
**3**	Ich grüble über Dinge.	I worry about things.
**4**	Ich fühle mich selten deprimiert (bedrückt).	I seldom feel blue.
**5**	Ich bin leicht zu beunruhigen.	I am easily disturbed.
**6**	Ich bin leicht aus der Fassung zu bringen.	I get upset easily.
**7**	Ich wechsle oft meine Stimmung.	I change my mood a lot.
**8**	Ich habe häufig Stimmungsschwankungen.	I have frequent mood swings.
**9**	Ich bin leicht zu reizen.	I get irritated easily.
**10**	Ich fühle mich oft deprimiert (bedrückt).	I often feel blue.
**Factor 2: extraversion**		
**11**	Ich bringe eine Party in Schwung.	I am the life of the party.
**12**	Ich spreche nicht viel.	I don't talk a lot.
**13**	Ich fühle mich wohl unter Menschen.	I feel comfortable around people.
**14**	Ich halte mich im Hintergrund.	I keep in the background.
**15**	Ich beginne Gespräche.	I start conversations.
**16**	Ich habe wenig zu sagen.	I have little to say.
**17**	Ich spreche mit vielen verschiedenen Leuten auf Partys.	I talk to a lot of different people at parties.
**18**	Ich mag es nicht, Aufmerksamkeit auf mich zu ziehen.	I don't like to draw attention to myself.
**19**	Ich habe kein Problem im Zentrum der Aufmerksamkeit zu stehen.	I don't mind being the center of attention.
**20**	Ich bin still unter Fremden.	I am quiet around strangers.
**Factor 3: openness to experience**		
**21**	Ich besitze einen großen Wortschatz.	I have a rich vocabulary.
**22**	Ich habe Schwierigkeiten abstrakte Ideen zu verstehen.	I have difficulty understanding abstract ideas.
**23**	Ich habe eine lebhafte Vorstellungskraft.	I have a vivid imagination.
**24**	Ich bin an abstrakten Ideen nicht interessiert.	I am not interested in abstract ideas.
**25**	Ich habe ausgezeichnete Ideen.	I have excellent ideas.
**26**	Ich habe kein gutes Vorstellungsvermögen.	I do not have a good imagination.
**27**	Ich verstehe Dinge schnell.	I am quick to understand things.
**28**	Ich gebrauche schwierige Wörter.	I use difficult words.
**29**	Ich verbringe Zeit damit über Dinge nachzudenken.	I spend time reflecting on things.
**30**	Ich bin voller Ideen.	I am full of ideas.
**Factor 4: agreeableness**		
**31**	Ich empfinde wenig für andere.	I feel little concern for others.
**32**	Ich bin interessiert an anderen Menschen.	I am interested in people.
**33**	Ich beleidige andere.	I insult people.
**34**	Ich fühle mit anderen Menschen mit.	I sympathize with others' feelings.

**35**	Ich bin nicht interessiert an den Problemen anderer Leute.	I am not interested in other people's problems.
**36**	Ich habe ein weiches Herz.	I have a soft heart.
**37**	Ich bin nicht wirklich interessiert an anderen.	I am not really interested in others.
**38**	Ich nehme mir Zeit für andere.	I take time out for others.
**39**	Ich fühle die Gefühle anderer.	I feel others' emotions.
**40**	Ich bringe Leute dazu, sich wohl zu fühlen.	I make people feel at ease.
**Factor 5: conscientiousness**		
**41**	Ich bin immer vorbereitet.	I am always prepared.
**42**	Ich lasse meine Sachen herumliegen.	I leave my belongings around.
**43**	Ich achte auf Details.	I pay attention to details.
**44**	Ich verursache großes Durcheinander.	I make a mess of things.
**45**	Ich erledige lästige Routinearbeiten unmittelbar.	I get chores done right away.
**46**	Ich vergesse oft, Dinge an ihren Platz zurückzulegen.	I often forget to put things back in their proper place.
**47**	Ich mag Ordnung.	I like order.
**48**	Ich drücke mich vor meinen Pflichten.	I shirk my duties.
**49**	Ich folge einem Plan.	I follow a schedule.
**50**	Ich bin anspruchsvoll in meiner Arbeit.	I am exacting in my work.

We used 50 items from the German translation of the IPIP (International Personality Item Pool, which consists of lexical Big-Five factor markers). In the task, sentences were changed to the third person singular (e.g., “she gets stressed out easily”).
