# Supplementary material for: Incorporating social knowledge structures into computational models
Source: Nat Commun. 2022 Oct 20;13:6205. doi: 10.1038/s41467-022-33418-2 (PMC9584930; doi:10.1038/s41467-022-33418-2)
Supplement: Supplementary file 3 — Reporting Summary [file 41467_2022_33418_MOESM3_ESM.pdf]

nature portfolio

Corresponding author(s): Koen M.M. Frolichs
Christoph W. Korn

Last updated by author(s): Jul 22, 2022

Reporting Summary

Nature Portfolio wishes to improve the reproducibility of the work that we publish. This form provides structure for consistency and transparency in reporting. For further information on Nature Portfolio policies, see our Editorial Policies and the Editorial Policy Checklist.

Statistics

For all statistical analyses, confirm that the following items are present in the figure legend, table legend, main text, or Methods section.

n/a	Confirmed	
☐	☒	The exact sample size (*n*) for each experimental group/condition, given as a discrete number and unit of measurement
☐	☒	A statement on whether measurements were taken from distinct samples or whether the same sample was measured repeatedly
☐	☒	The statistical test(s) used AND whether they are one- or two-sided<br>*Only common tests should be described solely by name; describe more complex techniques in the Methods section.*
☒	☐	A description of all covariates tested
☒	☐	A description of any assumptions or corrections, such as tests of normality and adjustment for multiple comparisons
☐	☒	A full description of the statistical parameters including central tendency (e.g. means) or other basic estimates (e.g. regression coefficient) AND variation (e.g. standard deviation) or associated estimates of uncertainty (e.g. confidence intervals)
☐	☒	For null hypothesis testing, the test statistic (e.g. *F*, *t*, *r*) with confidence intervals, effect sizes, degrees of freedom and *P* value noted<br>*Give P values as exact values whenever suitable.*
☒	☐	For Bayesian analysis, information on the choice of priors and Markov chain Monte Carlo settings
☐	☒	For hierarchical and complex designs, identification of the appropriate level for tests and full reporting of outcomes
☐	☒	Estimates of effect sizes (e.g. Cohen's *d*, Pearson's *r*), indicating how they were calculated

Our web collection on statistics for biologists contains articles on many of the points above.

Software and code

Policy information about availability of computer code

Data collection	The behavioral task was presented to human participants using the MATLAB toolbox Cogent (Version 1.32), on standard PCs with MATLAB version R2018b (The MathWorks Inc).
Data analysis	Behavioral data was analyzed using custom code in MATLAB, version R2018b (The MathWorks Inc). This code is freely available on Github (https://github.com/dnhi-lab/PerLe) and Zenodo (https://zenodo.org/record/4697286) [doi.org/10.5281/zenodo.4697286].

For manuscripts utilizing custom algorithms or software that are central to the research but not yet described in published literature, software must be made available to editors and reviewers. We strongly encourage code deposition in a community repository (e.g. GitHub). See the Nature Portfolio guidelines for submitting code & software for further information.

Data

Policy information about availability of data

All manuscripts must include a data availability statement. This statement should provide the following information, where applicable:

- Accession codes, unique identifiers, or web links for publicly available datasets
- A description of any restrictions on data availability
- For clinical datasets or third party data, please ensure that the statement adheres to our policy

The behavioral data that support the findings of this study are publicly available on Github (https://github.com/dnhi-lab/PerLe) and on Zenodo (https://zenodo.org/record/4697286) [doi.org/10.5281/zenodo.4697286].

Human research participants

Policy information about studies involving human research participants and Sex and Gender in Research.

Reporting on sex and gender	Sex and gender were not considered in the study design, i.e., we did not differentiate between participants (based on sex or gender) in our analyses. Sex was self-reported in a questionnaire before the study.
Population characteristics	See above
Recruitment	We recruited all participants via a local online platform. This platform is used by students, which was our intended study group.<br><br>The experiments were advertised as a standard social learning experiments. This might attract more socially inclined individuals who could bias our sample towards 'better' or faster social learning than one would expect from a standard student sample. However, the overall large sample over 5 experiments (with four independent samples) make us confident that such a bias would only play a minor role if any at all.
Ethics oversight	Ethik-Kommission der Ärztekammer Hamburg, Number: PV5746

Note that full information on the approval of the study protocol must also be provided in the manuscript.

Field-specific reporting

Please select the one below that is the best fit for your research. If you are not sure, read the appropriate sections before making your selection.

☐ Life sciences ☒ Behavioural & social sciences ☐ Ecological, evolutionary & environmental sciences

For a reference copy of the document with all sections, see nature.com/documents/nr-reporting-summary-flat.pdf

Behavioural & social sciences study design

All studies must disclose on these points even when the disclosure is negative.

Study description	Participants performed a social-learning task. Data are quantitative, i.e., trait-ratings for others.
Research sample	Participants for all of the five experiments consisted of students from local universities who were recruited via a local online platform. The research sample for each of the five experiments was ≥ 30 participants and thus comparable to behavioral learning studies inside (Rosenblau et al., 2018; Korn et al., 2012) and outside (Kang et al., 2021; Najar et al., 2020) our lab.<br><br>Exclusion criteria were focused on participants who did not finish (parts of) the task.<br><br>References<br>Rosenblau, G., Korn, C. W., & Pelphrey, K. A. (2018). A computational account of optimizing social predictions reveals that adolescents are conservative learners in social contexts. Journal of Neuroscience, 38(4), 974-988.<br><br>Korn, C. W., Prehn, K., Park, S. Q., Walter, H., & Heekeren, H. R. (2012). Positively biased processing of self-relevant social feedback. Journal of Neuroscience, 32(47), 16832-16844.<br><br>Kang, P., Burke, C. J., Tobler, P. N., & Hein, G. (2021). Why we learn less from observing outgroups. Journal of neuroscience, 41(1), 144-152.<br><br>Najar, A., Bonnet, E., Bahrami, B., & Palminteri, S. (2020). The actions of others act as a pseudo-reward to drive imitation in the context of social reinforcement learning. PLoS biology, 18(12), e3001028.
Sampling strategy	Sampling was based on participants' replies to our online recruitment. Sample sizes in all experiments were similar to standards in the field (≥ 30 participants, see citations above).
Data collection	Each participant gave informed consent before data collection. Data was collected using a task on a computer. Besides participants and researchers, no other person was present during data collection. The researchers were not blind to the study hypothesis. Conditions did not differ between participants such that all participants received the same instructions and treatment.
Timing	Pilot data were collected in April 2017. Data for all experiments were collected from April 2018 to July 2019.
Data exclusions	For Experiment 4, 1 participant was excluded and for Experiment 5, 2 participants were excluded. All exclusions were due to missing answers on > 10% of the total number of trials.
Non-participation	None of the invited participants dropped out or declined participation.

Randomization	Experiments were conducted sequentially, this meant we would only recruit participants for one experiment at a time (Experiments 4 & 5 were conducted on the same day with the same participant sample). Participants were only allowed to partake in one of the experiments (or both experiments 4 & 5).<br><br>Conditions never differed between participants in any of the experiments. Within participants, conditions (i.e., trials) were randomly ordered.<br><br>We did not specifically consider covariates because our experiments were about general personality learning on unknown profiles. That is, we do not expect any covariates (e.g., biases based on group, ethnicity, occupation) to have a large or even any effect.

Reporting for specific materials, systems and methods

We require information from authors about some types of materials, experimental systems and methods used in many studies. Here, indicate whether each material, system or method listed is relevant to your study. If you are not sure if a list item applies to your research, read the appropriate section before selecting a response.

Materials & experimental systems

n/a	Involved in the study	
☒	☐	Antibodies
☒	☐	Eukaryotic cell lines
☒	☐	Palaeontology and archaeology
☒	☐	Animals and other organisms
☒	☐	Clinical data
☒	☐	Dual use research of concern

Methods

n/a	Involved in the study	
☒	☐	ChIP-seq
☒	☐	Flow cytometry
☒	☐	MRI-based neuroimaging
